# An initial loading-dose vitamin D versus placebo after hip fracture surgery: randomized trial

**DOI:** 10.1186/s12891-016-1174-9

**Published:** 2016-08-11

**Authors:** Jenson CS Mak, Rebecca S. Mason, Linda Klein, Ian D. Cameron

**Affiliations:** 1 John Walsh Centre for Rehabilitation Research, Sydney Medical School Northern, University of Sydney, Sydney, New South Wales Australia; 2 Department of Physiology, School of Medical Sciences, The University of Sydney, Sydney, NSW Australia; 3 Office of Medical Education, Sydney Medical School, University of Sydney, Sydney, NSW Australia; 4 Faculty of Health and Medicine, The University of Newcastle, Newcastle, NSW Australia

**Keywords:** Hip fracture, Vitamin D, Randomized controlled trial, Falls, Rehabilitation

## Abstract

**Background:**

Improving vitamin D (25-OHD) status may be an important modifiable factor that could reduce disability severity, fall-rates and mortality associated after hip fracture surgery. Providing a loading-dose post-surgery may overcome limitations in adherence to daily supplementation.

**Method:**

In this randomized, double-blind, placebo-controlled trial, 218 adults, aged 65-years or older, requiring hip fracture surgery were assigned to receive a single loading-dose of cholecalciferol (250,000 IU vitamin-D3, the REVITAHIP - Replenishment of Vitamin D in Hip Fracture strategy) or placebo, both receiving daily vitamin-D(800 IU) and calcium (500 mg) for 26-weeks. Outcome measures were 2.4 m gait-velocity, falls, fractures, death (Week-4), 25-OHD levels, quality-of-life measure (EuroQoL) and mortality at weeks-2, 4 and 26.

**Results:**

Mean age of 218 participants was 83.9(7.2) years and 77.1 % were women. Baseline mean 25-OHD was 52.7(23.5)nmol/L, with higher levels at Week-2 (73 vs 66 nmol/L; *p* = .019) and Week-4 (83 vs 75 nmol/L; *p* = .030) in the Active-group, but not at Week-26. At week-4, there were no differences in 2.4 m gait-velocity (0.42 m/s vs 0.39 m/s, *p* = .490), fractures (2.7 % vs 2.8 %, *p* = .964) but Active participants reported less falls (6.3 % vs 21.1 %, χ^2^ = 4.327; *p* = 0.024), with no significant reduction in deaths at week-4 (1 vs 3, *p* = 0.295), higher percentage reporting ‘no pain or discomfort’ (96.4 % vs 88.8 %, *p* = 0.037), and trended for higher EuroQoL-scores (*p* = 0.092) at week-26. One case of hypercalcemia at week-2 normalised by week-4.

**Conclusion:**

Among older people after hip fracture surgery, the REVITAHIP strategy is a safe and low cost method of improving vitamin-D levels, reducing falls and pain levels.

**Trial registration:**

The protocol for this study is registered with the Australian New Zealand Clinical Trials Registry ANZCTRN ACTRN12610000392066 (Date of registration: 14/05/2010).

## Background

Hip fractures and related disabilities are important public health issues for older people around the world [1–6]. Despite the age-adjusted hip fracture rates reducing in countries such as Australia and the United States, the actual numbers of fractures are increasing steadily due to the increasing proportion of the elderly population. By 2040, an estimated 512,000 hip fractures will occur in the United States each year at a cost of $16 billion per year, [2] and by 2050, an estimated 76.7 billion Euros will be spent on this problem in Europe [3]. Outcomes for people who survive hip fracture are of concern, with more than one-quarter dying within a two-year period [7], and most not recovering their previous level of function [4, 5]. More than 10 % of survivors will be unable to return to their previous residence. Most of the remainder will have some residual pain or disability [6]. Given that people require assistance to recover from a hip fracture, personal and societal costs are often incurred following surgery due to the need for rehabilitation, outpatient visits for follow-up treatment, temporary residential aged care facility placement if required, and assistance with activities of daily living at home during the recovery period [8]. Given these factors, the quest in improving function after a hip fracture has the potential to be of enormous benefit to elderly people by reducing disability and improving quality of life. This can then reduce direct treatment costs and costs of long-term community or residential aged care services. The ability to mobilise is the key activity underlying functioning and quality of life [9].

Hypovitaminosis D is commonly associated with hip fracture in older people. It occurs because of several factors including decreased sun exposure with reduced skin production of vitamin D and low dietary D2/D3 intake. Vitamin D replacement, mostly with calcium, has been used successfully to reduce such fractures, as well as falls among older people [10–12]. Without preventive treatment, however, hypovitaminosis D following hip fracture may result in proximal muscle weakness, pain, reduced dynamic balance and performance speed, [13] affecting mobilization during the acute postoperative and rehabilitation periods [14–16]. This study assessed the efficacy and safety of a loading dose of cholecalciferol for the improvement of gait velocity, 25-hydroxycholecalciferol (25-OHD) levels, falls, fractures, functional outcomes and mortality in women and men who had undergone recent surgical repair of a hip fracture over a 26 week period.

## Methods

### Study design

The Replenishment of Vitamin D in Patients with Hip Fracture (REVITAHIP) Trial was a multicenter, randomized, double-blind, placebo-controlled trial involving patients with recent hip fracture [17]. Patients were randomly assigned to receive either an oral loading dose of cholecalciferol (at a dose of 250,000 IU vitamin D3) or placebo, both receiving daily supplementation with oral vitamin D (800 IU) and calcium (500 mg).^1^ Deviation from this protocol occurred for two participants (one in each group) with an initial serum 25-hydroxyvitamin D level of 10 nmol/L or less due to the known risks. These cases received an additional 14-day loading dose of vitamin D3 (at a dose of 4000 IU given orally), continuing on as per other patients for the remainder of the trial.

Patients were monitored for up to 26 weeks with telephone interviews or clinic/home visits at 2, 4, 12 (telephone interview only) and 26 weeks. All study procedures were approved by the local institutional review board (Northern Sydney Local Health District (NSLHD) - HREC Number 10/ HARBR/14). The study is registered with both the Australian Clinical Trial Registry (ACTR No. 12610000392066). This research was carried out in compliance with the Helsinki Declaration.

The academic investigators initiated the concept of the study. A data and safety monitoring board consisting of site investigators and the chief investigator met quarterly to oversee the conduct and safety of the study. Data analysis was performed by a statistician at the Office of Medical Education, Sydney Medical School, University of Sydney, New South Wales, Australia.

### Patients

All patients who were enrolled in the trial had undergone hip fracture surgery at one of three hospitals in Sydney and the Central Coast, New South Wales, Australia [17, 18]. They were invited to participate if they were aged 65 years or over and able to provide informed consent, either directly or via the person legally responsible for making decisions on their behalf, were deemed suitable by the treating medical team, and were able to take a loading dose of vitamin D within seven days after surgery [17]. Patients with delirium or dementia were included only after consent had been obtained from both the patient and the legal surrogate. The mobility and functional status of participants have been previously reported [18].

Exclusion criteria were: being bed-bound prior to fracture, or having a life expectancy deemed less than 1 month by the treating clinical staff; hypercalcemia (serum calcium >2.65 mmol/L); history of nephrolithiasis; thyrotoxicosis; Paget’s disease; sarcoidosis; malignancy (except skin cancer) and associated pathological fractures; significant renal impairment (serum creatinine >0.15 mmol/L); liver disease (alanine aminotransferase or aspartate aminotransferase level >2 times the upper limit of the normal range); undergoing treatment with calcitriol, or already undergoing treatment with > =1000 IU daily oral vitamin D3.

Mean (SD) age of participants was 83.9 (7.2) years and 77.1 % were women. Most participants (81.7 %) were born in Australia and 93.6 % spoke English as the first language. Approximately 83 % of participants had a pre-injury residence in the community, with 44 % living alone [18].

### Randomization and blinding

Patients were randomly assigned to treatment groups at a central location, accessed through a central telephone number and using a computer-generated random number schedule with variable block sizes of 2 to 6. Study patients, investigators, steering committee members, and faculty who adjudicated the clinical and safety end points remained unaware of study-group assignments throughout the trial.

### End points

The primary outcome measure was gait velocity over 2.4 m [19] and was measured at weeks 2, 4, and 26, with baseline assumed to be 0 immediately following surgery. Secondary outcome measures were the number of falls, fractures and hospitalizations, activities of daily living (using the Barthel Index [20]), quality of life (EQ5D [Euroqol] [21]), 25-OHD and calcium levels, grip strength, adherence to calcium and vitamin D supplements, and adverse events (including cardiovascular events and death).

### Assessment of outcomes (Table 1)

**Table 1:** REVITAHIP Outcome Measures

Outcome Measure	Time Measurement
2.4 m gait-velocity	Baseline, Weeks 2, 4
Grip strength	Baseline, Weeks 2, 4
Falls	Baseline, Weeks 2, 4
Fractures	Baseline, Weeks 2, 4
Mortality	Baseline, Weeks 2, 4
25-OHD levels	Baseline, Weeks 2, 4, 26
Quality-of-life measure (EuroQoL)	Baseline, Weeks 2, 4, 12, 26

Falls were recorded at 2 and 4 weeks using calendars where participants lived in the community, or using residential aged care facility or hospital records where participants were in care. A fall was defined according to the Kellogg definition [22] as an incident in which the body unintentionally comes to rest on the ground or other lower level which was not as a result of a violent blow, loss of consciousness, and sudden onset of paralysis as in a stroke or an epileptic seizure. Where a fall was recorded, determination according to this definition would follow via interview with the patient or their carer.

Fractures and hospitalizations (measured at 2 and 4 weeks) were recorded by the participants if living in the community or by care facility records and were verified by contact with the participant’s general practitioner or hospital. Quality of life was assessed at 4, 12 and 26 weeks using the EQ5D, a valid and reliable measure of quality of life in older people [21]. 25-OHD levels were determined using the DiaSorin assay [23] from the same laboratory for the three sites and measured at initial assessment, 2, 4, and 26 weeks. Grip strength in kilograms was assessed at initial assessment, 2, 4, and 26 weeks using a portable dynamometer (JAMAR hydraulic Hand Dynamometer manufactured by Sammons Prestons: Access Health. Unit 1 Rear, 194–196 Whitehorse Rd, BLACKBURN VIC 3130 Australia). Calcium/vitamin D adherence was recorded at initial assessment, 2, 4, 12 and 26 weeks by participants or their carer if living in the community or confirmed by care facility records. Rate of adherence was expressed as a percentage of doses taken up to each measurement point.

### Adverse events and laboratory measures

The site investigator reported adverse events and serious adverse events at each measurement point. Such events were categorized with the use of the Medical Dictionary for Regulatory Activities [24]. The expert committee whose members were unaware of the study-group assignment adjudicated laboratory criteria, adverse events and primary cause of death.

### Statistical analysis

Calculations were based on statistical power of 80 % with the alpha set at .05 (2-sided test). To address the primary hypothesis of the study, a sample size of 125 per group was initially estimated to show a 10 % difference in mean gait velocity improvement at the 4-week follow-up assessment. The number of 250 was not recruited due to feasibility of funding of 218 participants. Data were coded to permit blinding to group allocation in the initial statistical analysis. Differences in the primary outcome measure between the two groups over time were analyzed using repeat-measures analysis of variance (GLM in IBM SPSS version 21.0). Separate analyses assessing change over time relative to baseline were performed at each follow-up point to maximize use of the data available. Mean gait velocity change scores relative to baseline at weeks 2, 4 and 26 follow up, as well as between follow-up points, were calculated. Similar methods were used for secondary outcomes where data were continuous. Pearson chi-square test was used where outcome measures were categorical, supplemented with use of Fisher’s exact Chi square test when cell counts were small. Missing data at week 2 were imputed for one case with otherwise complete data using the within case mean of baseline and week 4 data. Imputation of missing data at other follow up points was not possible. Analyses followed the intention-to-treat principle. The study protocol specified that a 25-OHD assay be performed prior to the loading dose. However, despite requests prior to the loading dose, in 62 (28.4 %) cases the 25-OHD assay was conducted within 24–48 hours after the loading dose. These 62 participants were excluded from analyses using baseline 25-OHD as an independent or dependent variable. These 62 participants were, however, included in all other analyses. Logistic regression was used to determine the odds ratio for having a fall within the study period.

## Results

### Baseline characteristics and follow-up (Table 2)

**Table 2:** Summary Baseline Demographic and Clinical Characteristics of REVITAHIP Participants^a^

Variables	Active (*n* = 111)	Placebo (*n* = 107)	*P* value
Sex – n (%)			
- Female	84 (75.7)	84 (78.5)	0.633
- Male	27 (24.3)	23 (21.5)	
Country of Birth – n (%)			
- Australia	92 (82.9)	86 (80.4)	0.727
- Other	19 (17.1)	21 (19.6)	
Age			
Mean (years)^b^	83.7+/−7.5	84.1+/−7.0	0.726
Grouped – n (%)			
- 65-74 yr	15 (13.5)	10 (9.3)	0.772
- 75-84 yr	43 (38.7)	42 (39.3)	
- 85-94 yr	47 (42.3)	50 (46.7)	
- 95+ yr	6 (5.4)	5 (4.7)	
Body mass index (kg/m2)^b^	24.2+/−3.4	25.1+/−3.8	0.099
Number of days from admission to loading dose^b^	4.8+/−2.1	5.4+/−2.3	0.049
Pre-injury mobility – n (%)^d^			
- Fully independent	80 (72.1)	72 (67.9)	0.156
- Minimum help	15 (13.5)	15 (14.2)	
- Moderately help	8 (7.2)	7 (6.6)	
- Substantial help	1 (0.9)	8 (7.5)	
- Unable to perform task	7(6.3)	4(3.8)	
Pre-injury Modified Barthel index	87.5+/−19.9	86.9+/−22.2	0.852
Pre-injury Functional Comorbidity Index ^b^	3.37+/−2.1	2.9+/−1.7	0.063
Hip Fracture Subtype – n (%)^d^			
- Undisplaced subcapital	6 (5.7)	3 (2.8)	0.442
- Displaced subcapital:	50 (47.2)	48 (45.3)	
- Pertrochanteric simple (2-part)	5 (4.7)	5 (4.7)	
- Pertrochanteric complex (3-part)	27 (25.5)	34 (21.1)	
- Intertrochanteric/basicervical	5 (4.7)	9 (8.5)	
- Subtrochanteric	13 (12.3)	7 (6.6)	
Time from fracture to surgery (in hours)^b^	43.8+/−38.9	38.9+/−25.9	0.272
Hip Fracture Surgery^d^			0.655
- Cannulated screws	4 (3.7)	1 (0.9)	
- Uncemented hemiarthroplasty	15 (14.0)	14 (13.2)	
- Cemented hemiarthroplasty	8 (7.5)	9 (8.5)	
- Total hip replacement	19 (17.8)	20 (18.9)	
- Dynamic hip screw with short plate	14 (13.1)	8 (7.5)	
- Dynamic hip screw with long plate	19 (17.8)	20 (18.9)	
- Gamma nail	28 (26.2)	34 (32.1)	
MMSE score (mean, standard deviation)^b^	26.32+/−4.3	25.7+/−4.4	0.282
Total number of medications used^b^	5.1+/−2.6	4.9+/−2.6	0.201
25-hydroxyvitamin D level (nmol/L)^c^			
Mean	55.6+/−26.4	49.6+/−19.7	0.112
Grouped values: n (%)^e^			
- <30	13 (16.5)	11 (14.3)	0.621
- 30-49	22 (27.8)	27 (35.1)	
- > = 50	44 (55.7)	39 (50.6)	
Grip strength (kgs)^b^	16.4+/−7.3	15.7+/−6.2	0.478

^a^Plus-minus values are means +/− SD

^b^Continuous variables were compared with the use of a two-sample t-test. Categorical variables were compared with the use of a chi-square test

^c^For participants given the intervention loading dose after 25-OHD levels were taken (*n* = 156), (Placebo = 77; Active = 79)

^d^The following categories contained missing variables: hip fracture subtype (6 missing, *n* = 212), hip fracture surgery (6 missing, *n* = 212), premorbid mobility (1 missing, *n* = 217)

^e^Nowson CA, McGrath JJ, Ebeling PR, Haikerwal A, Daly RM, Sanders KM, Seibel MJ, Mason RS; Working Group of Australian and New Zealand Bone and Mineral Society, Endocrine Society of Australia and Osteoporosis Australia. Vitamin D and health in adults in Australia and New Zealand: a position statement. Med J Aust. 2012;196(11):686–7 [25]

**Fig. 1:**
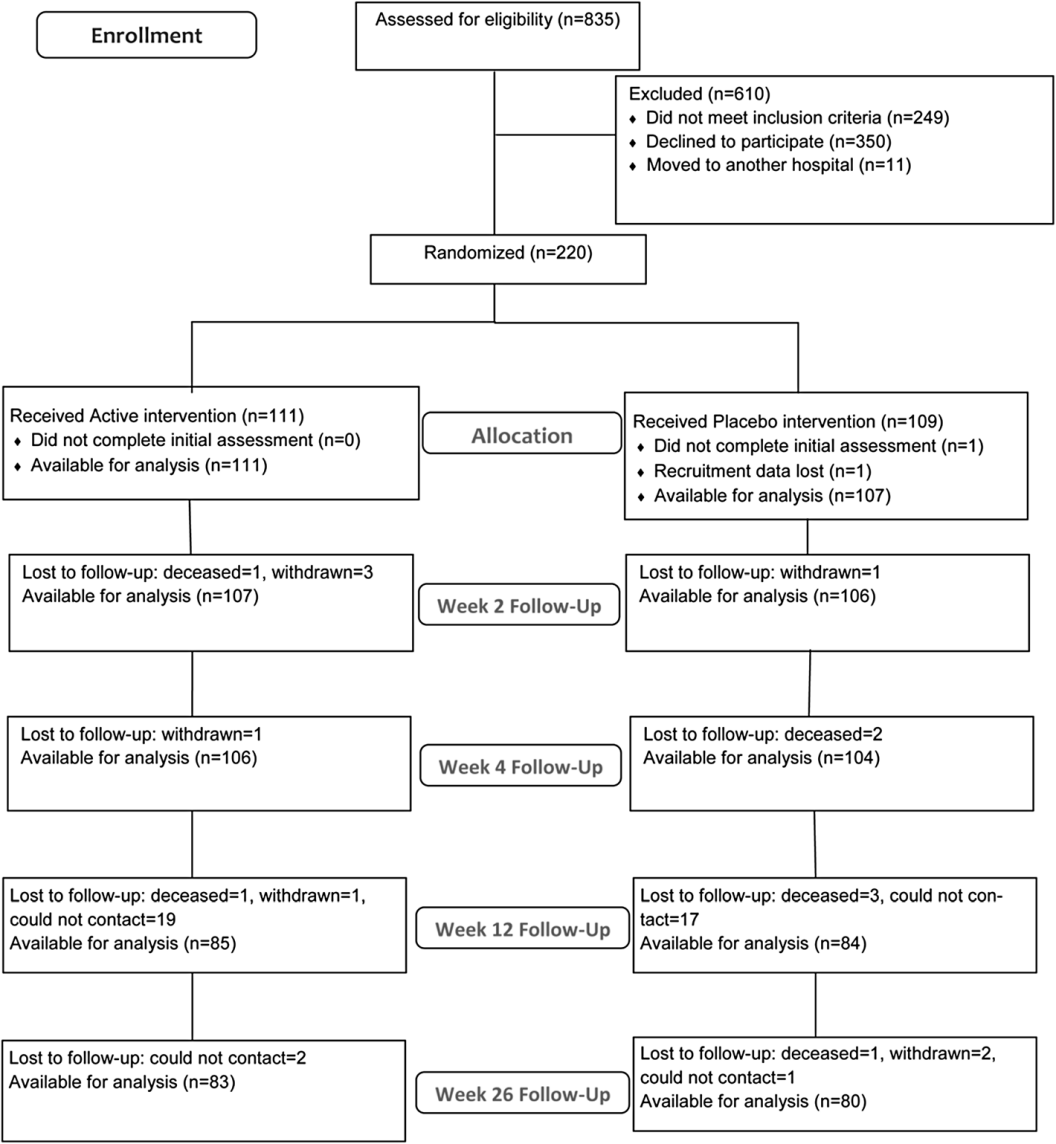
Enrolment and Flow of the Patients from REVITAHIP

Of a total of 218 patients, 111 patients were randomly assigned to receive loading dose cholecalciferol (Active) and 107 patients were assigned to receive placebo; 74.1 % of the patients completed the trial, whilst 95.5 % were successfully followed up to primary outcome at week 4 (Fig. 1). The median follow-up time was 24.8 weeks. Overall 18 % of participants were lost to follow-up, predominantly due to an inability to contact patients by phone for assessment. The rate of loss was similar in the two groups. The percentage of patients who reported adherence of at least 80 % or better to vitamin D and calcium supplements was 95.7 % at week 4, 95.9 % at week 12 and 96.3 % at week 26. Adherence did not differ significantly between the groups at any assessment point.

Most baseline demographic and clinical characteristics did not differ significantly between the two group with three exceptions. A higher percentage of the Active group reported comorbid diabetes (25.2 % vs 9.4 %; *p* = 0.002) and depression (21.6 % vs 8.5 %; *p* = 0.008) compared to the Placebo group. Also time from admission to loading dose was marginally less in the Active group than the Placebo group (4.8 vs 5.4 days, *p* = 0.049). The most common coexisting medical conditions in this population at baseline were arthritis, osteoporosis, upper gastrointestinal disease, visual impairment and diabetes [18].

### Gait velocity

Mean 2.4 m gait velocity at weeks 2, 4 and 26, as well as change scores between assessment points, are shown in Table 3. Patients across both groups showed consistent significant improvements over time. As expected, at week 4 both groups increased significantly in mean gait velocity to a combined mean of 0.404+/−0.30 m/s from a baseline of 0 (*p* = 0.000). However, there were no significant differences between the groups in improvements over time. Specifically, mean improvement in gait velocity at 4 weeks from baseline in the Active group was 0.419+/−0.295 m/s compared with 0.389+/−0.325 m/s in the Placebo groups (*p* = 0.490).

**Table 3:** Changes in gait velocity as a function of time and treatment group

	Active	N	Placebo	n	Significance^a^
Change in Gait Velocity					
- Baseline to week 2	0.173 +/−0.259	107	0.155 +/−0.255	106	0.608
- Baseline to week 4	0.419 +/−0.295	106	0.389 +/−0.325	104	0.490
- Baseline to week 26	0.753 +/−0.264	83	0.738 +/−0.258	80	0.718
- Week 2 to Week 4	0.250 +/−0.238	106	0.232 +/−0.249	104	0.582
- Week 2 to Week 26	0.549 +/−0.307	83	0.568 +/−0.282	80	0.694
- Week 4 to Week 26	0.316 +/−0.300	83	0.359 +/−0.356	80	0.404

^a^Significance level is shown for the time by group interaction (note for comparisons to baseline of 0, group and group by time interactions are identical). All changes over time across both groups reached significance at *p* = 0.000

### 25-hydroxyvitamin D (25-OHD) levels (Fig. 2)

**Fig. 2:**
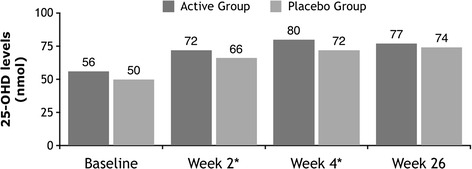
Mean 25-hydroxyvitamin D levels over 26 weeks (* significance noted at Week 2 and 4)

For patients in which 25-OHD levels were assessed prior to the intervention loading dose (*n* = 156), mean 25-OHD was 52.7+/−23.5 mmol/L (median = 54.2). Hypovitaminosis D (<50 nmol/L) was present in 46.8 % of these participants. Vitamin D levels among participants have been reported in an earlier paper where it was noted that 15.4 % of participants had levels <30 nmol/L and only 2 participants had levels <10 nmol/L.(36) Among these 156 participants there was a significantly higher percentage with adequate 25-OHD (> = 50 nmol/L) at week 4 (96.8 vs 84.6 %, *p* = 0.031) in the Active group when compared to the Placebo group. At week 2 and week 4 mean 25-OHD levels were significantly higher for the Active group versus the Placebo group (at week 2, 72+/−20. vs 66+/−19 nmol, *p* = 0.019; and at week 4, 80+/−20 vs 72+/−23 nmol/L, *p* = 0.049), but not at week 26. However, the groups did not differ significantly in the changes over the time intervals from baseline to weeks 2, 4, or 26.

### Falls and fractures

**Fig. 3:**
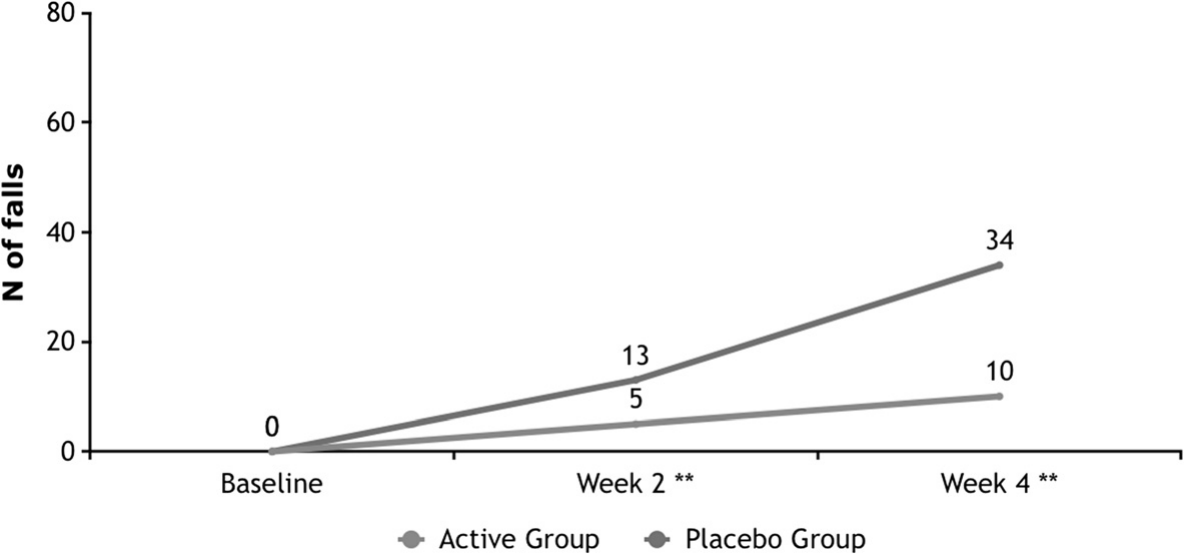
Cumulative number of falls over 4 weeks (** denotes statistical difference)

**Fig. 4:**
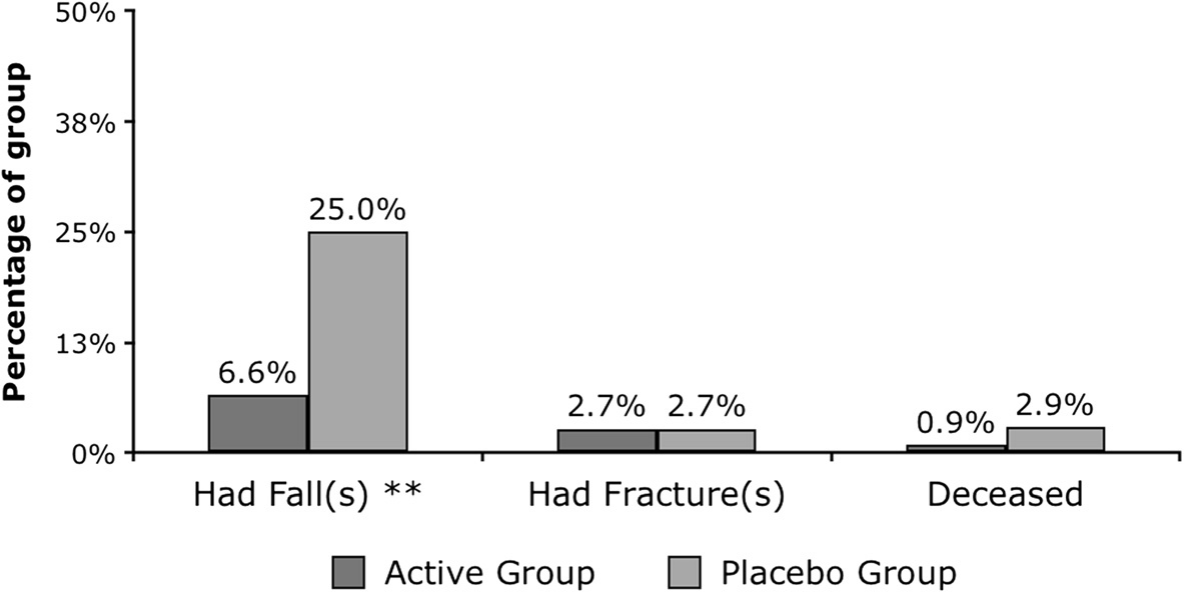
Fall, Fractures and Deaths over 4 weeks by Intervention Group (** denotes statistical difference)

A total of 44 new falls occurred in 30 patients by week four. To week 4, seven (6.3 %) participants in the Active group reported 1 or more falls compared to twenty-three (21.1 %) in the Placebo group (χ^2^ = 4.327; *p* = 0.024). At week 4, the Active group was associated with a falls rate of 250.0 (number of falls/days x 1000), as compared with 821.4 in the Placebo group; an absolute risk reduction of 57.1 % and a relative reduction of 69.6 % (Figs. 3 and 4). The odds ratio of any fall, for the Placebo group in comparison to the Active group, within the first 4 weeks of the study was 3.332 (CI: 1.340 to 8.235, *p* = 0.010). At week 4, three (2.7 %) of participants in the Active group had 1 or more fractures compared with 3 (2.8 %) in the Placebo group (*p* = .964). The risk reduction was very similar in the intention-to-treat and per-protocol populations. Further, at week 2, there was trend for lower 25-OHD (65.4 vs 71.8 nmol/L, *F* = 3.669, *p* = 0.057) among participants that fell, and at week 4, 25-OHD levels were significantly lower for those that fell (70.0 vs 82.1 nmol/L, *F* = 10.458, *p* = 0.001). There was a trend for statistical significance in 25-OHD concentrations between those who sustained a fracture compared to those who did not at week 4 (70.4 vs 78.5 nmol/L, *F* = 2.781, *p* = 0.097).

### Function and quality of life

Among patients in the Active group, the mean Barthel Index was 88.0+/−16.6, compared with 86.9+/−17.2 (*p* = 0.62) in the Placebo group at 4 weeks. Mean differences in Barthel Index at 4 weeks from premorbid functioning in the Active group was 0.55 compared with 0.05 in the Placebo groups (*p* = 0.96). There were no significant differences at each of the time points between the groups. Further, regarding grip strength, there were also no significant differences between the two groups at week 4 (18.6+/−7.8 vs 18.7+/−8.3kgs, *F* = .055, *p* = .815).

The health related quality of life data were as follows. Overall, there was no significant difference in total EuroQoL at Week 4 but a higher total EuroQoL score was noted for Active participants (88.1+/−13.2 vs 84.3+/−15.8, *F* = 2.87, *p* = 0.092), although not significant. EuroQoL subscales between the Active and Placebo groups were similar at Week 4 or 26, except for the EuroQoL pain subscale. Participants in the Active group were more likely to have ‘no pain or discomfort’ at Week 26 following hip fracture surgery (96.4 % vs 88.8 %, *p* = 0.037).

### Safety analysis

In the safety analysis, 4 of 218 patients (1.8 %) died during the study, of whom 1 (0.9 %) were in the Active group and 3 (2.8 %) were in the Placebo group. The hazard ratio of 3.054 (95 % CI, 0.816 to 15.133; *p* = 0.295) indicated the observed difference was not significant. The numbers of death were small relating to a combination of cardiovascular morbidity, and sepsis-related morbidity. Due to the small numbers there was an inability to detect a significant difference. No serious adverse events occurred in the two groups. One patient in the Active group (0.3 %) and no patient in the Placebo group had adjudicated hypercalcemia (serum corrected calcium > 2.65 nmol/L). There were no significant differences in mean serum corrected calcium levels at each of the assessment points.

## Discussion

The REVITAHIP study has shown that in older patients following hip fracture surgery, a loading dose of 250,000 IU vitamin D3 (“Active”) compared with Placebo, followed by treatment with vitamin D (800 IU) and calcium (500 mg) daily in both groups, resulted in higher 25-OHD levels and a greater percentage with target ‘sufficient’ 25-OHD levels (>50 nmol/L, [25, 26]), with no significant differences in gait velocity at 4 weeks. A significantly reduced incidence of falls but not fractures was noted in the Active group compared with Placebo over four weeks. This is surprising because in the study cohort the baseline level of 25-OHD was higher than in other studies of patients with hip fracture [27]. Further, the differences in 25-OHD from initial measurement to weeks 2 and 4 respectively between the Active and Placebo groups, while significant, were not large.

Older patients with hip fracture experience increased morbidity, functional decline, and death, as well as increased use of health care services, and therefore represent an important population to investigate for advancing improvements in quality of life and function, and the prevention of falls and secondary fractures. Our study provides much needed data to help determine whether vitamin D replenishment strategies are effective in such patients with osteoporosis. Because the patients in our study were older and had more coexisting conditions and a higher risk of falls than patients in many clinical trials of treatment for osteoporosis, our findings contain helpful information for clinicians and for patients who have had a hip fracture.

Poor adherence to vitamin D therapy has been shown to compromise the efficacy of this treatment for fracture reduction and, therefore, to increase medical costs [28]. Such findings have been particularly notable in frail older adults [29]. Vitamin D deficiency is frequently observed in older patients and is associated with an increased risk of hypocalcemia when intravenous bisphosphonates are administered before a normal vitamin D level has been achieved [13]. Due to the very high rates of vitamin D deficiency noted in the HORIZON Recurrent Fracture study (observed in the first 385 patients [30]), the REVITAHIP investigators adopted a similar loading dose vitamin D followed by maintenance Vitamin D at 800 IU (and calcium) daily supplied to the participants (with an overall adherence of >80 %). The results were that almost all the participants in the Active treatment group reached target vitamin D (>50 nmol/L) at week 2 and 4 compared with the Placebo group.

The Active group showed a non-significant lower mortality rate compared with the Placebo group which is likely related to the study being underpowered to detect a difference, or due to random variation. However, from results of the Dubbo Osteoporosis Study [1] that all low-trauma fractures were associated with increased mortality risk for 5 to 10 years, and subsequent fracture was associated with increased mortality risk for an additional 5 years, a significant reduction in falls and in the Active REVITAHIP group is likely to confer a mortality reduction in this high-risk population. Further, data from a large meta-analysis of eight prospective studies (26018 men and women) suggest those with vitamin D deficiency (in the bottom quintile) were 1.57 times more likely to die compared with those in the top quintiles. [31] Finally, there is a strong signal for mortality reduction from high-dose vitamin D in severely deficient ICU patients in the hospital period but not 6 months, suggesting the mortality reduction benefit is likely to be within the first 21 days of administration [32].

The safety profile for high-dose loading dose of vitamin D indicated few areas of concern, consistent with previous findings [1]. There was one case of biochemical hypercalcemia. We did not find an increased incidence of renal adverse events. With the loading dose of 250,000 IU vitamin D in a group of subjects who had already sustained a hip fracture, our findings contrast with those of Sanders et al. who reported increased falls and fractures following a loading dose of 500,000 IU in healthy post-menopausal females [31].

An interesting point of discussion deserves mention. Whilst there was a significant difference in the 25-OHD levels between the Active and Placebo groups at the early stages of rehabilitation (Week 2 and Week 4), it was a surprise finding that a 6.3 and 7.7 nmol/L difference in these levels, with no difference in gait velocity could translate into significant reductions in falls rates. These results showed that the vitamin D levels did not increase after 4 weeks even in the Active group with the high dose of vitamin D. This might be because of the half-life of serum 25- hydroxyvitamin D (normally 3 weeks) according to Holick [33]. There was also a large effect with falls reduction rate (relative reduction of 69.6 % at Week 4) in the Active group compared with other studies [34, 35]. However our results may have been a chance finding or influenced by unidentified factors. Finally, there was a small but significant difference (0.6 days) between the delivery of the loading-dose vitamin D such that this was provided earlier in the Active compared to the Placebo group. Possible explanations of this include optimization of proximal muscle strength [14, 15] and dynamic balance parameters, [15] to enable earlier effective rehabilitation. Our results suggest that other factors will need further investigation in the future.

Our study had several limitations. The study patients were, on average, slightly younger and healthier than are patients with hip fracture in the general population (83 vs 84 years old), [36] as suggested by data regarding 1-year mortality. However, patients in our study ranged widely in age (up to 101 years), and some had cognitive impairment. Perhaps due to inclusion and exclusion criteria, there was under-recruitment of people with very low 25-OHD levels compared to the usual population with hip fractures due to the low number of participants from residential aged care facility (10 %) and participants with fewer total comorbidities. Our cohort of participants had relatively good function with moderate independence prior to their hip fracture. However, the investigators believe that this could also be considered a strength as it alerts clinicians to potential problems (e.g. in ADLs, mobility, high psychotropic medication use) even in a relatively well functioning cohort. Furthermore, it proved difficult to obtain an accurate diary of falls from all participants over the longer term. So falls data from weeks 12 and 26 could not be included in the presentation of the data. Indeed, our data contrasts with that from Vital-D study (which showed increased falls and fractures in the first 3 months of the study) [37]. Whilst Vital-D had a longer follow-up period for falls (3 years vs 4 weeks), there were vast differences in participant characteristics and study protocol with REVITAHIP, with the former study being (1) community-dwelling women only, (2) younger population (age 74 versus 84, by 10 years), (3) participants given yearly higher boluses of 500,000 IU for 3 years, and (4) participants not provided with any regular maintenance vitamin D/calcium therapy. Further, a higher percentage of the Active group reported comorbid diabetes compared to the Placebo group. Whilst there is a higher association of patient groups with osteoporosis who have diabetes, overall fracture rates in the Active group did not show a significance difference compared with the placebo group. Finally, our participants had a higher level of baseline 25-OHD compared to expected, likely owing to a less frail participant population. There is still significant discussion in the literature regarding the optimal 25OHD level for benefit, and a number of reports suggest progressive decline in fall risk at levels above 80 nmo/L [37], whilst others recommended level of 60 nmo/L in Summer [38]. Another limitation was that the planned sample size of 250 could not be achieved due to time and funding limitations in participant recruitment. Nevertheless, as 25-OHD levels in the REVITAHIP trial at baseline were higher than usual concentrations, and all patients received daily supplementation with vitamin D and calcium, the trial may have underestimated effects on falls, fractures and death. The rate of follow-up to final interview was lower than ideal at 74 %. Also, the length of follow-up for mortality was short (4 weeks) compared to other studies which have a longer follow-up time (5–10 years) [37]. It was the study team’s aim to complete a total follow-up of 26 weeks, but this was not possible. However, there was no evidence of differential loss to follow-up between the study groups. Next, the limited number of participants (*n* = 62) who had 25-OHD level measured prior to loading dose precluded a complete examination of the true value of baseline 25-OHD levels (leading to an over-estimation of 25-OHD levels in the groups. However, our intention-to-treat analyses on primary and secondary outcomes ensures that the results are accurate and true to the ‘a priori' hypotheses of the REVITAHIP study. Further, we understand that the significant finding regarding pain would not maintain significance if strict correction for multiple outcomes was undertaken (e.g. Bonferroni correction), however, we believe that the results are best interpreted for their clinical relevance and significance. Finally, the inclusion of a history of falls and osteoporotic fractures over the preceding 12 months, as well as a detailed dietary history of calcium and vitamin D and the degree of sun-exposure, parathyroid hormone levels and analyses examining their relationship with baseline vitamin D status would have strengthened the study.

## Conclusion

In conclusion, our findings indicate that treatment with 250000 IU cholecalciferol within 7 days after hip fracture surgery is associated with higher percentage of replete 25-OHD, reduced rates of falls and reduced pain levels. Given the relatively low expense of this intervention, and beneficial impact on the burden of morbidity and mortality from hip fracture, further evidence (in the form of longitudinal safety studies) is required to confirm the findings of our study.

## Abbreviations

25-OHD, 25-hydroxycholecalciferol [vitamin D]; EQ5D, Euroquol; HORIZON, Health Outcomes and Reduced Incidence with Zoledronic Acid Once Yearly; REVITAHIP, replenishment of vitamin D in hip fracture; Vital-D, ‘vitamin D’ study

## Footnotes

1 Since the protocol paper, there had been concerns regarding the safety of high-dose oral calcium supplements on participants on cardiovascular morbidity and mortality and thus the dosage of calcium was altered.
